# Different Glucose Metabolic Features According to Cancer and Immune Cells in the Tumor Microenvironment

**DOI:** 10.3389/fonc.2021.769393

**Published:** 2021-12-13

**Authors:** Hongyoon Choi, Kwon Joong Na

**Affiliations:** ^1^ Department of Nuclear Medicine, Seoul National University Hospital, Seoul, South Korea; ^2^ Department of Nuclear Medicine, Seoul National University College of Medicine, Seoul, South Korea; ^3^ Department of Thoracic and Cardiovascular Surgery, Seoul National University Hospital, Seoul, South Korea; ^4^ Department of Thoracic and Cardiovascular Surgery, Seoul National University College of Medicine, Seoul, South Korea

**Keywords:** glucose metabolism, glucose transporter, tumor microenvironment, immunotherapy, single cell RNA sequencing

## Abstract

**Background:**

A close metabolic interaction between cancer and immune cells in the tumor microenvironment (TME) plays a pivotal role in cancer immunity. Herein, we have comprehensively investigated the glucose metabolic features of the TME at the single-cell level to discover feasible metabolic targets for the tumor immune status.

**Methods:**

We examined expression levels of glucose transporters (GLUTs) in various cancer types using The Cancer Genome Atlas (TCGA) data and single-cell RNA-seq (scRNA-seq) datasets of human cancer tissues including melanoma, head and neck, and breast cancer. In addition, scRNA-seq data of immune cells in the TME acquired from human melanoma after immune checkpoint inhibitors were analyzed to investigate the dynamics of glucose metabolic profiles of specific immune cells.

**Results:**

Pan-cancer bulk RNA-seq showed that the GLUT3-to-GLUT1 ratio was positively associated with immune cell enrichment score. The scRNA-seq datasets of various human cancer tissues showed that GLUT1 was highly expressed in cancer cells, while GLUT3 was highly expressed in immune cells in TME. The scRNA-seq data obtained from human melanoma tissues pre- and post-immunotherapy showed that glucose metabolism features of myeloid cells, particularly including GLUTs expression, markedly differed according to treatment response.

**Conclusions:**

Differently expressed GLUTs in TME suggest that GLUT could be a good candidate a surrogate of tumor immune metabolic profiles and a target for adjunctive treatments for immunotherapy.

## Introduction

Dysfunction of the immune system in the tumor microenvironment (TME) plays a crucial role in cancer progression. Interaction of various cells in the TME with cancer cells causes immune suppression and/or exhaustion to induce cancer cellular progression (1, 2). Regarding tumor metabolism, impaired immune function due to metabolic competition between cancer and immune cells is one of the critical factors of an immunosuppressive TME (3, 4). Specifically, aerobic glycolysis, which is a typical metabolic feature of many malignant cells (the so-called ‘Warburg effect’) is also the primary energy source for activating immune cells (5). Therefore, cancer cells gain aggressive features by surpassing nutrient consumption to inhibit immune cell metabolism (6). The close interaction of tumor immune and metabolic profiles provides an opportunity to target the metabolism of the TME to enhance the efficacy of cancer immunotherapy and develop a diagnostic and prognostic biomarker for evaluating metabolic immune functionality (7).

Cancer shows a broad spectrum of immune profiles and metabolic properties in the TME, which eventually results in heterogeneity (8, 9). Thus, the metabolic profiles of the TME can be used for investigating the immune functionality of tumors (10). Metabolic reprogramming in cancer and immune cells due to cancer immunotherapy and/or chemoradiotherapy is a dynamic process that causes a variable response to immunotherapy (7, 11, 12). In this regard, a previous investigation showed the reciprocal change of glucose transporter (GLUT) between cancer and immune cells in lung cancer (13). GLUT1 and GLUT3 were enriched in lung cancer cells and immune cells of the TME, respectively (13). Furthermore, as PD-1 signaling inhibits glycolysis in T cells and PD-L1 in cancer cells stimulates aerobic glycolysis, this reciprocal glucose uptake associated with the PD-1-PD-L1 axis can be applied to metabolic modulation with immune checkpoint inhibitors (ICIs) as a new strategy for cancer immunotherapy (14, 15). Therefore, investigation of the TME metabolic profiles in various cancer subtypes and dynamic metabolic changes associated with ICIs are needed for developing a biomarker reflecting metabolism and a novel therapeutic strategy.

In this study, we investigated the glucose metabolism profiles based on RNA transcripts of various cells in the TME. First, we tested whether differently enriched GLUTs could be a surrogate of metabolic competition across multiple cancer types using The Cancer Genome Atlas (TCGA) and single-cell RNA-sequencing (scRNA-seq) datasets from different human cancer tissues. In addition, glucose metabolic profiles of various cells in the TME were analyzed according to the ICI response using the publicly available scRNA-seq data. Hence, we aimed to investigate the feasibility of glucose metabolic profiles as a biomarker reflecting immune metabolic functionality in the TME and to understand the metabolic dynamics of various cells affected by ICIs. A better understanding of the glucose metabolism of the TME at the single-cell level can facilitate the development of novel biomarkers for immunotherapy and therapeutics targeting cancer immunometabolism.

## Materials and Methods

### Data

We used multiple datasets of scRNA-seq and spatial transcriptome as well as bulk RNA-seq data of TCGA. Datasets used in this study is summarized in **Supplementary Table 1**.

### Preprocessing for Transcriptomic Data of TCGA

Using the ‘Recount2’ R package (16), we downloaded the RNA sequence data of 32 solid cancers obtained with the Illumina HiSeq 2000 mRNA platform (Illumina, San Diego, CA, USA). The coverage counts were scaled to estimate read counts using the *scale_counts* function of the recount2 package. For normalization, the read counts of all genes were converted into counts-per-million. We merged the transcriptome data of 32 solid cancers of TCGA projects into one large-scale expression matrix for pan-cancer analysis.

### Immune Cell Enrichment Analysis and Hypoxia Score

To evaluate overall immune cell enrichment in the TME, cell type enrichment scores were evaluated. A gene-signature based method, the xCell tool (http://xcell.ucsf.edu/), for inferring cell types from tissue transcriptome profiles was used (17). It infers 64 immune and stromal cell types of the TME. The composite score of immune cells (i.e., the ImmuneScore) was obtained for the analysis. More specifically, the immune score was defined as the sum of cell enrichment scores of B cells, CD4+ T-cells, CD8+ T-cells, dendritic cells, eosinophils, macrophages, monocytes, mast cells, neutrophils, and NK cells estimated by xCell. To estimate hypoxia score from RNA-seq data, single sample gene set enrichment analysis (ssGSEA) using hypoxia gene signature of biocarta pathway was applied (18).

### scRNA-Seq Analysis

The scRNA-seq data were scaled to log-normalization after the read counts were divided by the total number of transcripts and multiplied by 10,000. Two thousand highly variable genes were selected using the *FindVariableFeatures* function of Seurat (version 3.0) (19) based on a variance stabilizing transformation. The data were then scaled to z-scores. Principal component analysis was run on variable genes, and 10 principal components were selected for clustering analyses. The graph-based clustering approach was implemented using the *FindClusters* function. A key parameter to determine the number of clusters is the conservative resolution, which was set according to each dataset: 1.0 for the scRNA-seq data of breast cancer; 0.3 for the scRNA-seq data of HNSC; 0.2 for the scRNA-seq data of GBM; 0.5 for the scRNA-seq data of human melanoma treated with ICIs.

The scRNA-seq data were embedded by two-dimensional projection using t-SNE. To identify the marker genes of the clusters, the *FindAllMarkers* function of Seurat was used, and the first five high-ranked marker genes were identified according to the fold-change. The marker genes that were relatively highly expressed in a given cluster were extracted using the nonparametric Mann–Whitney U test.

Cancer cells and immune cells were identified by known marker genes of specific cell types as well as the extracted markers. For each dataset, at least four cell types were identified using specific well-known markers: cancer cells (EPCAM for epithelial cell origin tumors), T-cells (CD3D, CD8A, and CD4), B-cells (CD79A and IGHM), and myeloid cells (CD68 and LYZ). To identify cancer cells of GBM, we utilized chromosomal copy number alterations as described in previous studies (20, 21). Specifically, expression levels of genes located on chromosome 7 were extracted for all cells, and the scores for copy number alterations were calculated using a module score. The module score was evaluated using the *AddModuleScore* in the Seurat package. The cluster with a high copy number alteration score was regarded as a cancer cell cluster.

### Spatial Transcriptomic Data Analysis

Spatial transcriptome data obtained from the 10X Genomics Visium platform were downloaded from publicly available datasets of 10X Genomics (https://www.10xgenomics.com/resources/datasets/). A human breast cancer dataset with spatial information, and H&E staining was used. The data were preprocessed with ‘*SCTransfrom’* function of Seurat version 3. Immune cell enrichment scores were evaluated by xCell as described in the aforementioned section.

As a single gene feature count is sparse, we extracted module scores of GLUT1 and GLUT3 using each gene feature’s correlated genes. The GLUT1 and GLUT3 module scores were calculated using top-k positively correlated genes sorted by the Pearson’s correlation (k = 50). Gene ontology (GO) of GLUT1 (and GLUT3) correlated genes was evaluated using clusterProfiler (22). The module scores for each spot were calculated by *AddModuleScore* in the Seurat package.

### Differentially Expressed Genes of Single Cell Clusters and Functional GO Functional Enrichment Analysis

To compare a specific cluster of scRNA-seq data with other clusters, we used the *FindMarkers* function of the Seurat package. Differentially expressed genes of the cluster were selected according to the following criteria: absolute log fold change > 0.25 and false discovery rate (FDR) corrected p-value < 0.05. GO functional pathway analysis was performed using the clusterProfiler (22). The GO terms were filtered with a cut-off of p-value < 0.05 and FDR of less than 0.2.

### Calculating Enrichment Scores of Gene Functional Pathways

To examine the overall activities of glycolysis and OXPHOS of the TME, we used Reactome to select genes of certain metabolic pathways (23). The curated gene sets of the Reactome glycolysis and OXPHOS pathways to define metabolic profiles were obtained from MSigDB (Broad Institute, version 6.0). We used the *AddModuleScore* function of Seurat to calculate the scores of glucose metabolic pathways.

### Statistical Analysis

The correlations between variables were evaluated using Pearson’s correlation analysis. Comparison of the expression levels of gene features or module scores of two different clusters was performed using the Mann–Whitney U test. Further, comparison of the expression levels of multiple clusters was performed using the Kruskal–Wallis test. A p-value of less than 0.05 was considered statistically significant. All statistical analyses were performed using the R software package, version 4.0.2. (http://www.R-project.org).

## Results

### Different Expression of GLUTs Represents Immune Enrichment in Various Solid Tumors

**Figure 1:**
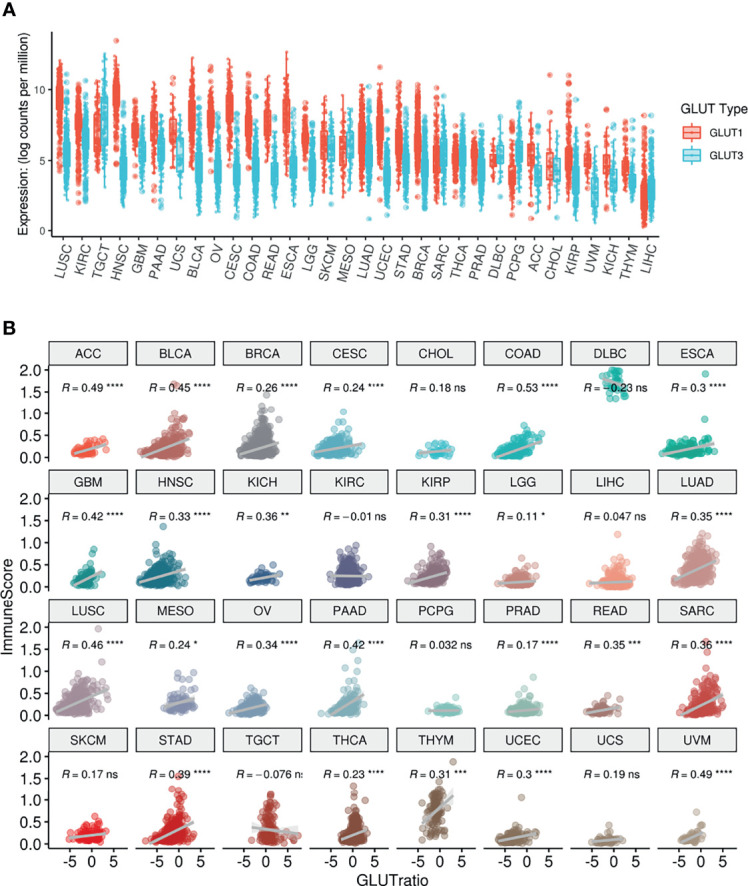
Pan-cancer analysis of GLUT expression and their association with ImmuneScore. **(A)** The distribution of GLUT1 and GLUT3 according to TCGA cancer types. **(B)** ImmuneScore of the TME and its association with GLUTratio (GLUT3/GLUT1). The positive correlation between ImmuneScore and GLUTratio was observed in most cancer types. ACC, Adrenocortical carcinoma; BLCA, Bladder Urothelial Carcinoma; BRCA, Breast invasive carcinoma; CESC, Cervical squamous cell carcinoma and endocervical adenocarcinoma; CHOL, Cholangiocarcinoma; COAD, Colon adenocarcinoma; DLBC, Lymphoid Neoplasm Diffuse Large B-cell Lymphoma; ESCA, Esophageal carcinoma; GBM, Glioblastoma multiforme; HNSC, Head and Neck squamous cell carcinoma; KICH, Kidney Chromophobe; KIRC, Kidney renal clear cell carcinoma; KIRP, Kidney renal papillary cell carcinoma; LGG, Brain Lower Grade Glioma; LIHC, Liver hepatocellular carcinoma; LUAD, Lung adenocarcinoma; LUSC, Lung squamous cell carcinoma; MESO, Mesothelioma; OV, Ovarian serous cystadenocarcinoma; PAAD, Pancreatic adenocarcinoma; PCPG, Pheochromocytoma and Paraganglioma; PRAD, Prostate adenocarcinoma; READ, Rectum adenocarcinoma; SARC, Sarcoma; SKCM, Skin Cutaneous Melanoma; STAD, Stomach adenocarcinoma; TGCT, Testicular Germ Cell Tumors; THCA, Thyroid carcinoma; THYM, Thymoma; UCEC, Uterine Corpus Endometrial Carcinoma; UCS, Uterine Carcinosarcoma, UVM, Uveal Melanoma. (ns, not significant; *p < 0.05; **p < 0.01; ***p < 0.001; ****p < 0.0001).

The expression levels of GLUT1 and GLUT3, which are the main transporters for glucose uptake in cancer cells (24), were compared across different types of cancers using TCGA data. Most cancers showed higher levels of GLUT1 than GLUT3 expression, while testicular germ cell tumors, mesothelioma, sarcoma, diffuse large B-cell lymphoma, thyroid carcinoma, pheochromocytoma/paraganglioma, and liver hepatocellular carcinoma showed higher levels of GLUT3 than GLUT1 expression (**Figure 1A**). According to a previous study on lung cancer, GLUT3 is relatively highly expressed in immune cells within the TME, while GLUT1 is highly expressed in most cancer cells (13). Thus, we suggested that the GLUT3-to-GLUT1 ratio (GLUTratio) could reflect immune cell metabolism compared with cancer cell metabolism. We performed a correlation analysis between the GLUTratio and the composite immune enrichment score (ImmuneScore) across all cancer types. As a result, most cancer types showed a significant positive correlation between the GLUTratio and ImmuneScore (**Figure 1B**). Among the 32 cancer types, 24 presented with a significant moderate and positive correlation. Notably, an insignificant negative correlation was found in diffuse large B-cell lymphoma, which originated from immune cells. This positive association between the GLUTratio and immune profiles was also found between the GLUTratio and cytolytic score, which was calculated through granzyme B and perforin A expression levels to reflect effector T-cell functionality (25) (**Supplementary Figure 1**). As it is well known that GLUT1 is upregulated in hypoxia, we evaluated the association between GLUTratio and the hypoxia signature. While the hypoxia signature was positively correlated with GLUT1 expression in most cancer types, GLUTratio was not (**Supplementary Figure 2**).

### Single-Cell Level Analyses Reveal Different Patterns of GLUT Expression in the TME of Various Cancers

**Figure 2:**
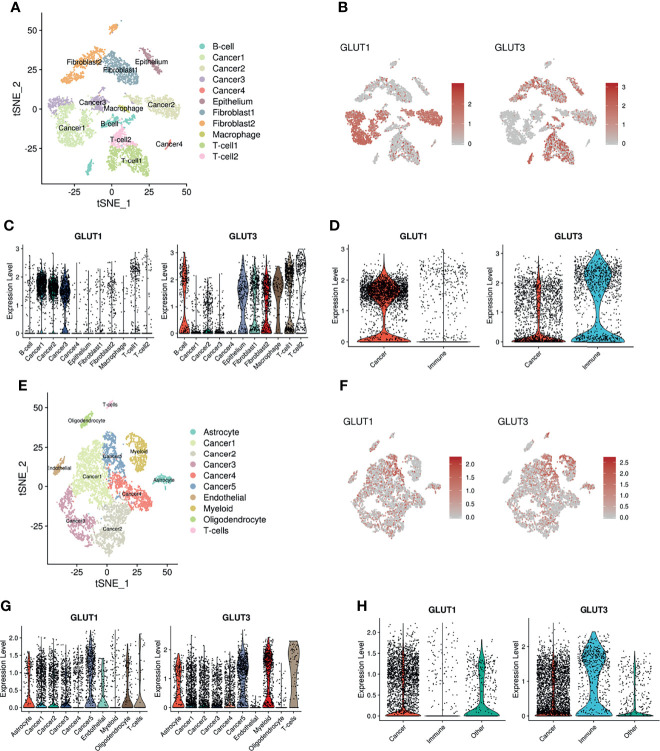
Distribution of GLUT1 and GLUT3 within the TME of HNSC and GBM. **(A)** Two-dimensional visualization of scRNA-seq data of HNSC by t-SNE analysis. **(B)** t-SNE plot indicating different expression patterns of GLUT1 (*left*) and GLUT3 (*right*) across cancer and other cell clusters including immune cells. **(C)** Violin plots showing the expression distribution of GLUT1(*left*) and GLUT3 (*right*) across cancer and other cell clusters of HNSC. **(D)** Violin plots showing GLUT1 (*left*) and GLUT3 (*right*) expression in cancer and immune cell clusters of HNSC. GLUT1 expression was higher in cancer cells, while GLUT3 expression was higher in immune cells. **(E)** Two-dimensional visualization of scRNA-seq data of GBM by t-SNE analysis. **(F)** t-SNE plot indicating different expression patterns of GLUT1 (*left*) and GLUT3 (*right*) across cancer and other cell clusters. **(G)** Violin plots showing the expression distribution of GLUT1(*left*) and GLUT3 (*right*) across cancer and other cell clusters of GBM. **(H)** Violin plots showing GLUT1 (*left*) and GLUT3 (*right*) expression in cancer and immune cell clusters of GBM.

To confirm whether the different expression patterns of GLUTs represent immune profiles of the TME, we employed multiple scRNA-seq datasets from various human solid tumors. The scRNA-seq data of human head and neck squamous cell carcinoma (HNSC) (26) were clustered, and immune cells of the TME and cancer cells were identified (**Figure 2A**) using marker genes of each cluster (**Supplementary Figure 2**). Their expression patterns were then visualized using t-distributed stochastic neighborhood embedding (t-SNE) plots. GLUT1 and GLUT3 were differentially enriched according to clusters **(** **Figures 2B, C**). When clusters were categorized into two cell types (i.e., cancer and immune cells), GLUT3 and GLUT1 expression levels were significantly higher in immune cells and cancer cells, respectively (**Figure 2D**). Similar results were also found for other cancer types. The scRNA-seq data of human glioblastoma multiforme (GBM) were clustered (**Figure 2E**), and their marker genes are presented in **Supplementary Figure 3**. Cancer cell clusters were identified using the chromosomal copy number alteration score calculated by the gene expression of chromosome 7 (21) (**Supplementary Figure 3**). GLUT1 and GLUT3 also showed different patterns according to clusters (**Figures 2F, G**). As demonstrated in HNSC, GLUT1 and GLUT 3 were enriched in cancer cells and immune cells, respectively (**Figure 2H**). This different GLUT expression pattern in cancer and immune cells was also found in breast cancer (27) (**Supplementary Figure 4**). In addition, the hypoxia score was evaluated for scRNA-seq data of HNSC and GBM (**Supplementary Figure 5**). It showed that the hypoxia score was not specifically increased in a certain cell type such as cancer cell.

**Figure 3:**
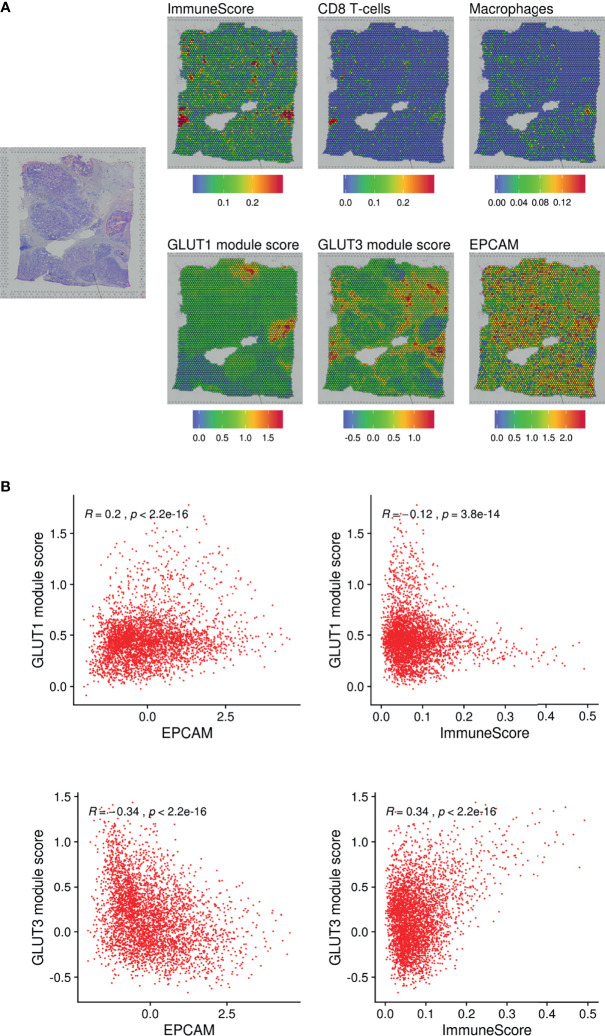
Spatial distribution of GLUTs and immune cells in the breast cancer tissue. **(A)** Gene expression features and module scores for GLUT1 and GLUT3-correlated genes were spatially mapped using spatial transcriptomic data of the breast cancer tissue. ImmuneScore, the enrichment score of CD8 T-cells and macrophages were estimated by xCell analysis. **(B)** Pearson correlation analyses were performed on GLUT1 and GLUT3 module scores with EPCAM expression and ImmuneScore. The correlation analyses were performed across spatially distributed spots on the breast cancer tissue.

We additionally analyzed a spatial transcriptome dataset obtained from human breast cancer tissue. Spatial gene features were represented using spatially resolved transcriptome data of 3,813 spots (**Figure 3A**). The module scores of GLUT1 and GLUT3 were assessed using each gene feature’s associated genes (**Supplementary Figure 6A**). The GO results of GLUT1-associated and GLUT3-associated genes showed GLUT1 was spatially associated with metabolic process such as NAD metabolic process, while GLUT3 was spatially associated with extracellular matrix and regulation of inflammation response (**Supplementary Figure 6B**). The GLUT3-associated module score was spatially correlated with ImmuneScore, CD8-T-cells, and macrophages (**Figures 3A, B**), while the GLUT1-associated module score was spatially positively correlated with EPCAM expression and negatively correlated with ImmuneScore (**Figures 3A, B**). As the positive correlation between the GLUTratio and ImmuneScore was revealed by the pan-cancer analysis with TCGA data, results of the scRNA-seq and spatial transcriptome data showed that GLUT3 was highly expressed among immune cells in the TME compared with that in cancer cells.

### ICIs Change GLUTs Expression and Glycolysis Score of Immune Cells

**Figure 4:**
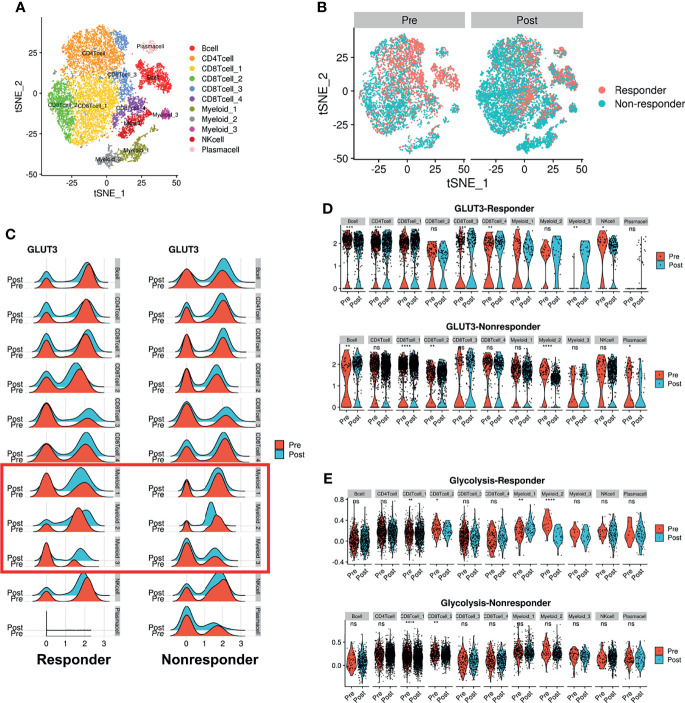
Dynamic change of GLUT3 and glycolytic activity in immune cells after ICI treatment in melanoma patients. **(A)** t-SNE plot showing scRNA-seq data obtained from melanoma patients before and after ICI treatment. **(B)** t-SNE plot of pre-treatment (*left*) and post-treatment (*right*) patients, color-coded by ICI response. **(C)** Ridge plots showing GLUT3 expression at pre-treatment and post-treatment across immune cell clusters according to the response to ICIs. A red box indicates myeloid cells that showed different patterns of GLUT3 expression after ICI treatment in accordance with the treatment response. **(D)** Violin plots showing the expression distribution of GLUT3 across immune cell clusters in responders and non-responders. **(E)** Violin plot showing glycolysis activity enrichment scores across immune cell clusters in responders and non-responders. (ns, not significant, *p < 0.05, **p < 0.01, ***p < 0.001, ****p < 0.0001; uncorrected p-values).

We further analyzed whether glucose metabolic profiles (including GLUTs) were dynamically changed according to ICI treatment since reciprocal glucose uptake between cancer and immune cells reflects tumor immune function (3, 13, 28). A scRNA-seq dataset of human melanoma cells from pre- and post-treatment with ICI was analyzed (29). CD45+ immune cells were clustered into 11 cell types (**Figure 4A**). Consistent with our previous findings, GLUT3 was upregulated in all immune cells compared with GLUT1 (**Supplementary Figure 7A**). To characterize the subsets of immune cell clusters, the marker genes of subsets were depicted in **Supplementary Figure 7B**. ‘CD8Tcell_1’ and ‘CD8Tcell_2’ were related to exhausted T-cells. ‘CD8Tcell_3’ represented TCF7+ memory/effector cells (29). ‘Myeloid_1’ and ‘Myeloid_2’ types represented macrophages and ‘Myeloid_3’ represented markers related to plasmacytoid dendritic cells (**Supplementary Figure 7B**). Immune cell populations were differently distributed according to the response to ICI and pre- and post-treatment (**Figure 4B**). First, we compared GLUT3 expression levels according to ICI treatment. The GLUT3 level was increased in specific immune cell types after ICI treatment, and the degree of increment was different according to the response to ICI (**Figure 4C**). In particular, GLUT3 of the CD8+ T-cell cluster, which is the key player in ICI (‘*CD8Tcell_1*’), was increased after ICI treatment in both responders and non-responders (**Figure 4D**). Notably, the change in GLUT3 level after ICI treatment was different between responders and non-responders in myeloid cells, instead of CD8 T-cells (**Figure 4D**). In particular, GLUT3 expression in a myeloid cell cluster, ‘*Myeloid_2*’, was decreased among non-responders only. Furthermore, changes in GLUT3 expression in myeloid cells after ICI treatment were largely heterogeneous between responders and non-responders.

Considering that effector immune cells depend on glycolysis for energy consumption, we additionally analyzed enrichment scores of glucose metabolism of immune cells. Myeloid cells showed higher glycolysis and oxidative phosphorylation (OXPHOS) scores than other immune cells (**Supplementary Figure 7C**). The glycolysis score of ‘*CD8 T-cell_1*’ was increased after ICI in both responders and non-responders. The glycolysis score was also remarkably different according to the response to ICI in myeloid cells as the GLUT3 expression level changes. The difference in glycolysis according to the ICI response was also prominent in myeloid clusters. A cluster, ‘*Myeloid_1*’, showed increased glycolysis and ‘*Myeloid_2*’ showed decreased glycolysis after ICI treatment in responders, while the glycolysis of both clusters was not changed in non-responders (**Figure 4E**).

### Metabolic Reconfiguration of Myeloid Cells Associated With ICI Response

**Figure 5:**
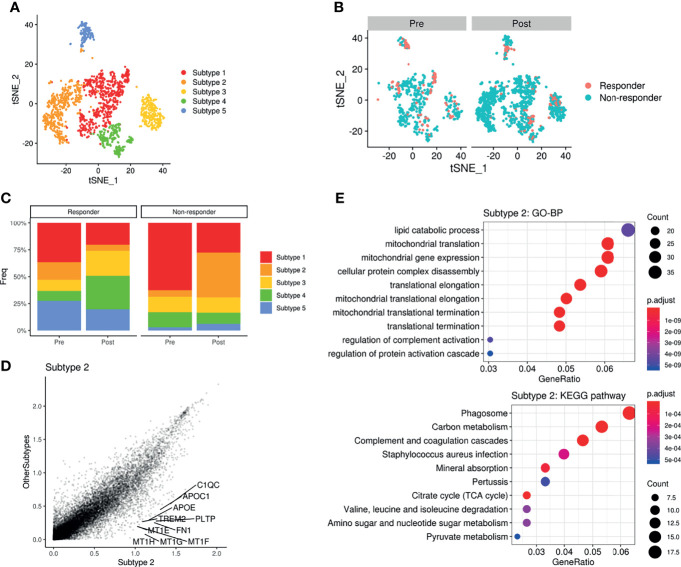
Metabolic remodeling of myeloid cells and a myeloid cluster increase in a non-responder to ICI. **(A)** t-SNE plot of the myeloid cell subset analyzed from the scRNA-seq data of melanoma patients before and after ICI treatment. **(B)** t-SNE plot of pre-treatment (*left*) and post-treatment (*right*) patients, color-coded by ICI response. **(C)** Barplots showing the distribution of myeloid subtypes in responders (*left*) and non-responders (*right*), before and after ICI treatment. Notably, ‘subtype2’ was remarkably increased in non-responders after ICI. **(D)** Scatterplot of the gene expression correlation between myeloid subtype 2 and other myeloid subtypes. Top 10 genes highly expressed in the subtype 2 cluster were labeled. **(E)** Dot plots showing the significant up-regulated GO terms of biological processes and KEGG pathways of myeloid subtype 2. The size of the dot is based on gene counts enriched in the pathway, and the color of the dot shows the significance of pathway enrichment.

As the glucose metabolic profiles of myeloid cells were significantly modified after ICI treatment, we further analyzed myeloid cell subsets for a more detailed examination of metabolic changes. The myeloid subsets were re-clustered (**Figure 5A**) and the landscape of scRNA-seq data, as visualized with t-SNE plots, varied a lot according to ICI treatment (**Figure 5B**). In particular, non-responders showed a remarkable increase in a subtype of the myeloid cluster (‘subtype 2’) after ICI, while this cluster was decreased in responders after ICI (**Figure 5C**). According to the differential gene expression analysis, the marker genes of ‘subtype 2’ were identified as PLTP, MT1G, APOC1, APOE, and MT1H (**Figure 5D**). These differentially expressed genes of ‘subtype 2’ myeloid cells were associated with the lipid catabolic process and mitochondrial functions in GO analysis (**Figure 5E**). According to the KEGG pathway analysis, these genes were associated with ‘phagosome’ as the most significantly associated pathway (**Figure 5E**). GLUTs were differently expressed in the ‘subtype2’ cluster among myeloid cell clusters where GLUT3 was highly expressed in the TME according to the previous results. The ‘subtype 2’ cluster showed relatively low GLUT3 and high GLUT1 expression levels compared with ‘subtype 1’, which is the most abundant myeloid cell subtype in the TME (**Supplementary Figures 7D, E**). The OXPHOS score of ‘subtype 2’ was the highest among the five myeloid clusters (**Supplementary Figures 7D, E**). The ‘subtype 2’ cluster also showed high expression of classical M2 macrophage markers (MRC1 and CD163), and it also expressed some M1 macrophage markers, including CD86, ITGAX, HLA-DRA, and STAT1. Furthermore, it included PD-L1-positive cells (**Supplementary Figure 7F**). Another type, ‘subtype 4’, was relatively increased in responders after ICI. The ‘subtype 4’ was associated with IDO1, CD1C, and NRDG2 expression as well as M1 markers (**Supplementary Figure 7G**). According to the subset analysis of myeloid cells, non-responders showed increased myeloid cells with high OXPHOS, relatively high GLUT1 expression, low GLUT3 expression, and PD-L1-positive types. Thus, this non-responder associated myeloid cell cluster was different from the immune cells in TME which relatively showed high GLUT3 and low GLUT1 according to our previous results.

## Discussion

In this study, we explored the glucose metabolic features of the TME at the single-cell level. First, differently expressed GLUTs between cancer and immune cells in the TME were found in various cancer types according to bulk, single-cell and spatial RNA-seq analyses. Second, we found that glucose metabolic profiles of immune cells in the TME were associated with ICI treatment response. In non-responders, specific subtypes of myeloid cells increased after ICI treatment were associated with high GLUT1, instead of GLUT3 expression, and M2 macrophage markers.

We proposed a new surrogate marker for immune functionality in terms of metabolism resulting from different GLUT expression in cancer and immune cells. The dynamic interactions of cancer cells with the TME affect local factors favoring cancer progression, and this may lead to immunosurveillance avoidance (1). One of the key mechanisms for cancer immune escape is metabolic competition between cancer and immune cells within a nutrient-deprived TME (3, 4). Despite this key mechanism of metabolic interaction, the development of biomarkers reflecting the functional metabolic status of the TME or new therapeutics targeting TME metabolism adjunctive to immunotherapy is still insufficient. We demonstrated that the GLUTratio (GLUT3-to-GLUT1 ratio) could be a feasible biomarker for predicting ICI response (13). In this study, we showed that the GLUTratio was positively correlated with ImmuneScore in most solid cancer types. scRNA-seq analysis showed a relatively higher expression of GLUT3 in immune cells in multiple cancer types, including HNSC, GBM, breast cancer, and lung cancer (13). Furthermore, for the breast cancer tissue, GLUT3 was spatially positively associated with immune-rich areas while GLUT1 was negatively associated. These findings suggest that different types of GLUTs in the TME could be a good common target for identifying immune functionality in various cancer types. Notably, GLUT1 was associated with the hypoxia signature, while GLUTratio was not. It supports that GLUTratio was different from hypoxia signature and more related to immune profiles of tumors. Furthermore, according to scRNA-seq data, the hypoxia signature was not specifically increased in a certain cell subtype such as cancer cell. Thus, it suggested that the specific upregulation of GLUT1 and GLUT3 in different cell types in TME was not associated with hypoxia. In addition, it could be applied to develop a therapeutic strategy combined with immunotherapy. As glucose uptake is mainly mediated by GLUT1 in cancer cells, selective inhibition of GLUT1 could suppress cancer cellular glucose metabolism (30). In addition, it can be hypothesized that relatively increased glucose uptake of immune cells due to metabolic competition could be expected to have a synergistic effect on immunotherapy. Previous preclinical studies have shown anti-cancer effects of selective GLUT1 inhibition (31–33); however, the effect of GLUT1 inhibition on the immune profiles in the TME has not been analyzed. Further studies regarding the adjunctive role of GLUT1 inhibition in immunotherapy are needed.

We then explored whether the glucose metabolic profiles of various cells in the TME were altered by ICI treatment. As the GLUTratio is associated with immune cell enrichment and cytolytic score, we expected increased molecular features related to glucose uptake and consumption in effector immune cells of the TME after ICI treatment. scRNA-seq data obtained from melanoma patients treated with ICIs revealed increased GLUT3 expression and glycolysis in CD8+ T-cells. However, increased GLUT3 expression and glycolysis in CD8+ T-cells were commonly found in both responders and non-responders. The increased glycolysis in CD8+ T-cells after ICI treatment was consistent with previous *in vitro* and animal studies that showed suppressed glycolysis due to PD1-PD-L1 interaction in T-cells (34, 35). PD-1 pathway blocking could increase the glycolysis of T-cells regardless of the response to ICIs. Instead, the major difference between responders and non-responders in terms of glucose metabolism was found in myeloid cells.

Our results on the metabolic alterations in myeloid cells in the TME according to the response to ICIs suggested the critical role of tumor-associated macrophages (TAMs) in ICI resistance. In particular, a specific cluster of myeloid cells increased after ICI treatment in non-responders. According to the analysis of human melanoma scRNA-seq, myeloid cells associated with responders showed a relatively high expression of *PLTP, APOC1, APOE, MT1G, and MT1H*. These genes were associated with lipid catabolic processes, carbon metabolism, and the TCA cycle. This cluster also showed a higher OXPHOS score, relatively low GLUT3 expression, and high GLUT1 expression. The specific TAM that increased after ICI treatment in non-responders is important to understand the role of TAM in the acquisition of resistance to ICI as well as to develop a biomarker beyond T-cells (36, 37). The myeloid cell cluster associated with non-responders showed moderate expression of GLUT1 and glycolysis genes and expression of M1 markers including CD86 and ITGAX, but with high expression of M2 markers, including MRC1 (CD206). In addition, KEGG pathway analysis showed that the most associated gene set was ‘phagosome’ activity. This implies that this subtype of myeloid cells could be associated with a previously suggested mechanism of ICI resistance, which is phagocytosis of antibody-blocking T-cell binding (36). The phagosome of TAM is induced by antibody-dependent cellular phagocytosis, followed by upregulation of PD-L1 in TAM, which consequently contributes to immunosuppression (38). Notably, the cluster associated with ICI resistance showed high PD-L1 expression according to our results. Additionally, the increased ICI-resistant myeloid cells with high phagosome activity, PD-L1 positivity, and moderate GLUT1/glycolysis expression. Another myeloid subtype with relatively high IDO1 and CD1c tended to be increased after ICI in responders, though IDO1 is associated with immunosuppression. It suggests that only some of the myeloid immunosuppressive changes may affect the ICI response, while other changes may not affect the response.

There are some limitations to this study that should be addressed. First, to explore the differential glucose metabolism within the TME, we investigated the expression values of GLUT and enrichment scores of metabolic pathways estimated from transcriptome data. Furthermore, precise measurement of cellular metabolism is difficult because enrichment scores cannot directly reflect functional protein activity. As an explorative study using multiple publicly available datasets of RNA-seq to identify targets of glucose metabolic profiles, further mechanistic and functional studies should be conducted for protein-level analysis and functional metabolic profiling to measure glucose consumption to validate and extend our study findings. Second, we used multiple RNA-seq datasets; therefore, different experimental protocols and preprocessing methods might affect the results. Finally, even though we analyzed multiple RNA-seq data from several different types of cancers, it might be difficult to generalize our findings to all cancer types. As different types of cancers have different compositions of immune and stromal cells in the TME, the responsiveness and the change in cellular composition by ICI might be different between cancer types.

We demonstrated that GLUTs are expressed differently in cancer and immune cells in the TME, and there are relatively high levels of GLUT3 in the immune cells of the TME, commonly in various cancer types. By analyzing scRNA-seq data obtained pre- and post-immunotherapy, we identified a specific myeloid subset characterized by high GLUT1 expression, PD-L1 positivity, phagosome activity, and markedly increased positive M2 markers in the non-responder group. This cluster could play a key role in ICI resistance. The characterization of glucose metabolism in cancer and immune cells within the TME might help in developing a biomarker reflecting tumor metabolism. Moreover, our findings provide insight for developing targets of glucose uptake to overcome ICI resistance as well as to find a combination of immunotherapy.

## Data Availability Statement

The original contributions presented in the study are included in the article/**Supplementary Material**. Further inquiries can be directed to the corresponding authors.

## Ethics Statement

Patient data were acquired using a publicly available dataset that removed patient identifiers. The publicly available data were collected with patients’ informed consent and approved by the Institutional Review Boards of all participating institutions in accordance with the 1964 Helsinki Declaration and its later amendments or comparable ethical standards.

## Author Contributions

HC and KJN designed the study, analyzed and interpreted the data, wrote and edited the manuscript, and read and approved the manuscript.

## Funding

This research was supported by the National Research Foundation of Korea (NRF) and funded by the Korean government (MSIT) (No. 2020R1C1C1007105, No. 2020M3A9B6037195, and No.2020M3A9B6038086), as well as the Korea Health Technology R&D Project through the Korea Health Industry Development Institute (KHIDI), and by the Ministry of Health & Welfare, Republic of Korea (No. HI19C0339).

## Conflict of Interest

HC is a cofounder and CTO of Portrai inc. and a scientific advisory board member for AItheNutriGene. KJN is a cofounder of Portrai, inc.

## Publisher’s Note

All claims expressed in this article are solely those of the authors and do not necessarily represent those of their affiliated organizations, or those of the publisher, the editors and the reviewers. Any product that may be evaluated in this article, or claim that may be made by its manufacturer, is not guaranteed or endorsed by the publisher.

## Abbreviations

TME, tumor microenvironment; GLUTs, glucose transporters; scRNA-seq, single-cell RNA-sequencing; ICIs, immune checkpoint inhibitors; TCGA, The Cancer Genome Atlas; HNSC, head and neck squamous cell carcinoma; t-SNE, t-distributed stochastic neighborhood embedding; GBM, glioblastoma multiforme; OXPHOS, oxidative phosphorylation; KEGG, Kyoto Encyclopedia of Genes and Genomes; TAM, tumor-associated macrophages.
