# Supplementary material for: Different Glucose Metabolic Features According to Cancer and Immune Cells in the Tumor Microenvironment
Source: Front Oncol. 2021 Dec 13;11:769393. doi: 10.3389/fonc.2021.769393 (PMC8710507; doi:10.3389/fonc.2021.769393)
Supplement: Supplementary file 1 [file DataSheet_1.pdf]

Supplementary Material

1 Supplementary Figures and Tables

1.1 Supplementary Figures

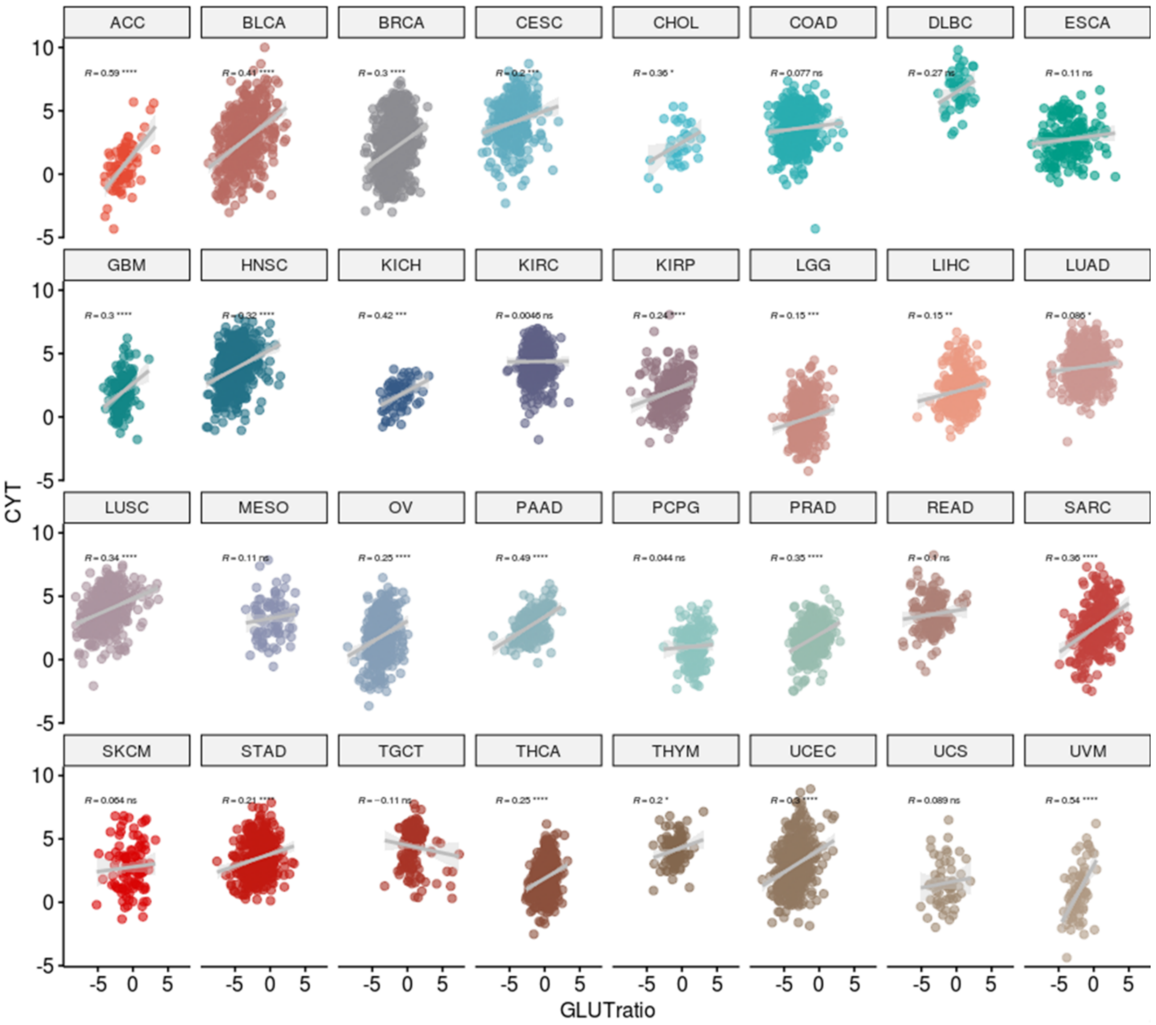

Supplementary Figure 1. The association between GLUTratio and cytolytic score across TCGA cancer types. The positive correlation between the cytolytic score and GLUTratio was observed in most cancer types. (ACC: Adrenocortical carcinoma; BLCA: Bladder Urothelial Carcinoma; BRCA: Breast invasive carcinoma; CESC: Cervical squamous cell carcinoma and endocervical adenocarcinoma; CHOL: Cholangiocarcinoma; COAD: Colon adenocarcinoma; DLBC: Lymphoid Neoplasm Diffuse Large B-cell Lymphoma; ESCA: Esophageal carcinoma; GBM: Glioblastoma multiforme; HNSC: Head and Neck squamous cell carcinoma; KICH: Kidney Chromophobe; KIRC: Kidney renal clear cell carcinoma; KIRP: Kidney renal papillary cell carcinoma; LGG: Brain Lower

Grade Glioma; LIHC: Liver hepatocellular carcinoma; LUAD: Lung adenocarcinoma; LUSC: Lung squamous cell carcinoma; MESO: Mesothelioma; OV: Ovarian serous cystadenocarcinoma; PAAD: Pancreatic adenocarcinoma; PCPG: Pheochromocytoma and Paraganglioma; PRAD: Prostate adenocarcinoma; READ: Rectum adenocarcinoma; SARC: Sarcoma; SKCM: Skin Cutaneous Melanoma; STAD: Stomach adenocarcinoma; TGCT: Testicular Germ Cell Tumors; THCA: Thyroid carcinoma; THYM: Thymoma; UCEC: Uterine Corpus Endometrial Carcinoma; UCS: Uterine Carcinosarcoma: UVM: Uveal Melanoma; CYT: Cytolytic Score)

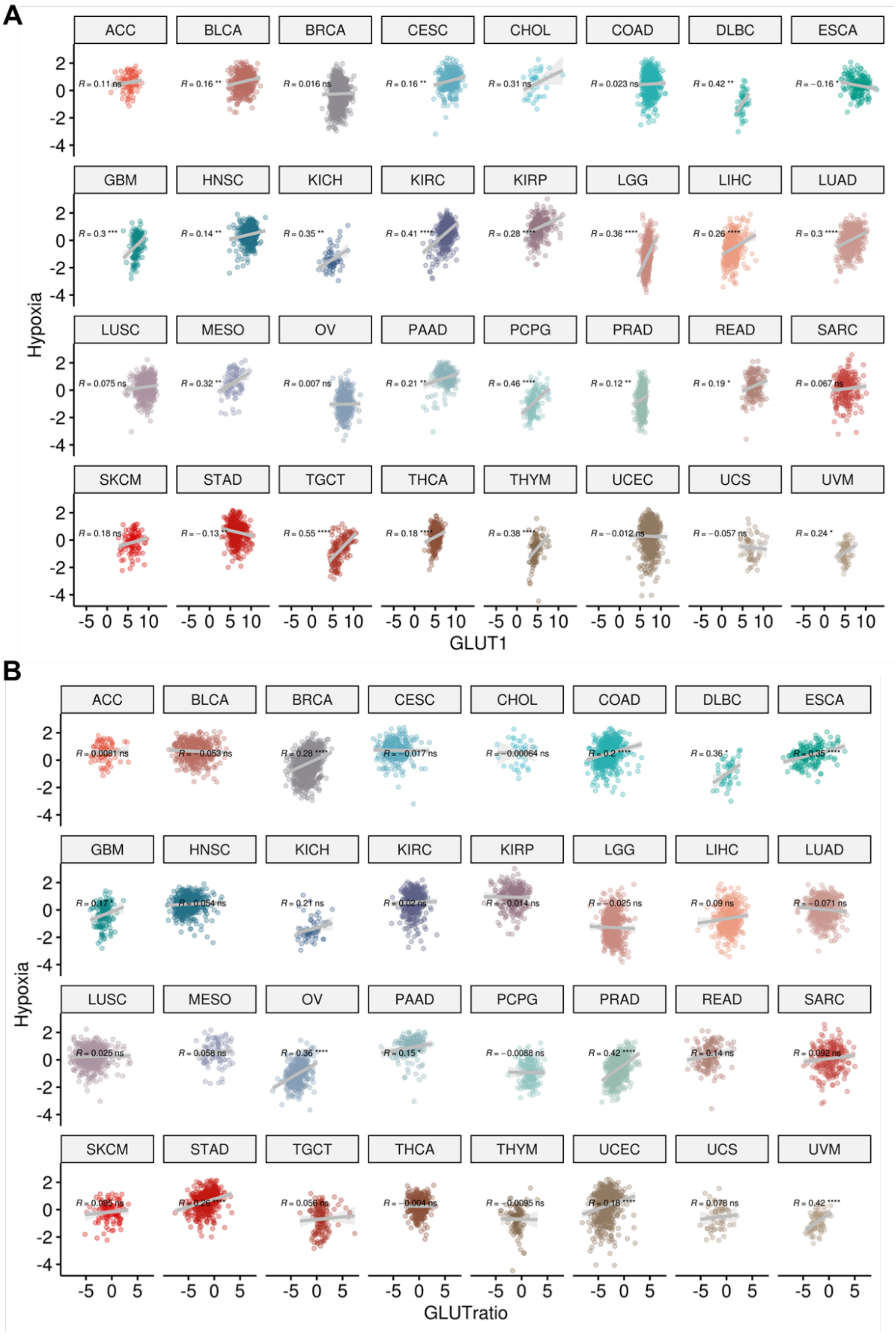

Supplementary Figure 2. The association between hypoxia and GLUTratio across TCGA cancer types. (A) The positive correlation between the hypoxia score and GLUT1 expression was observed in most cancer types. (B) However, the hypoxia score was not correlated with GLUTratio in

most cancer types, while GLUTratio of a few cancer types (including BRCA, COAD, ESCA, OV, PRAD, STAD, and UVM) showed weak positive correlation with hypoxia.

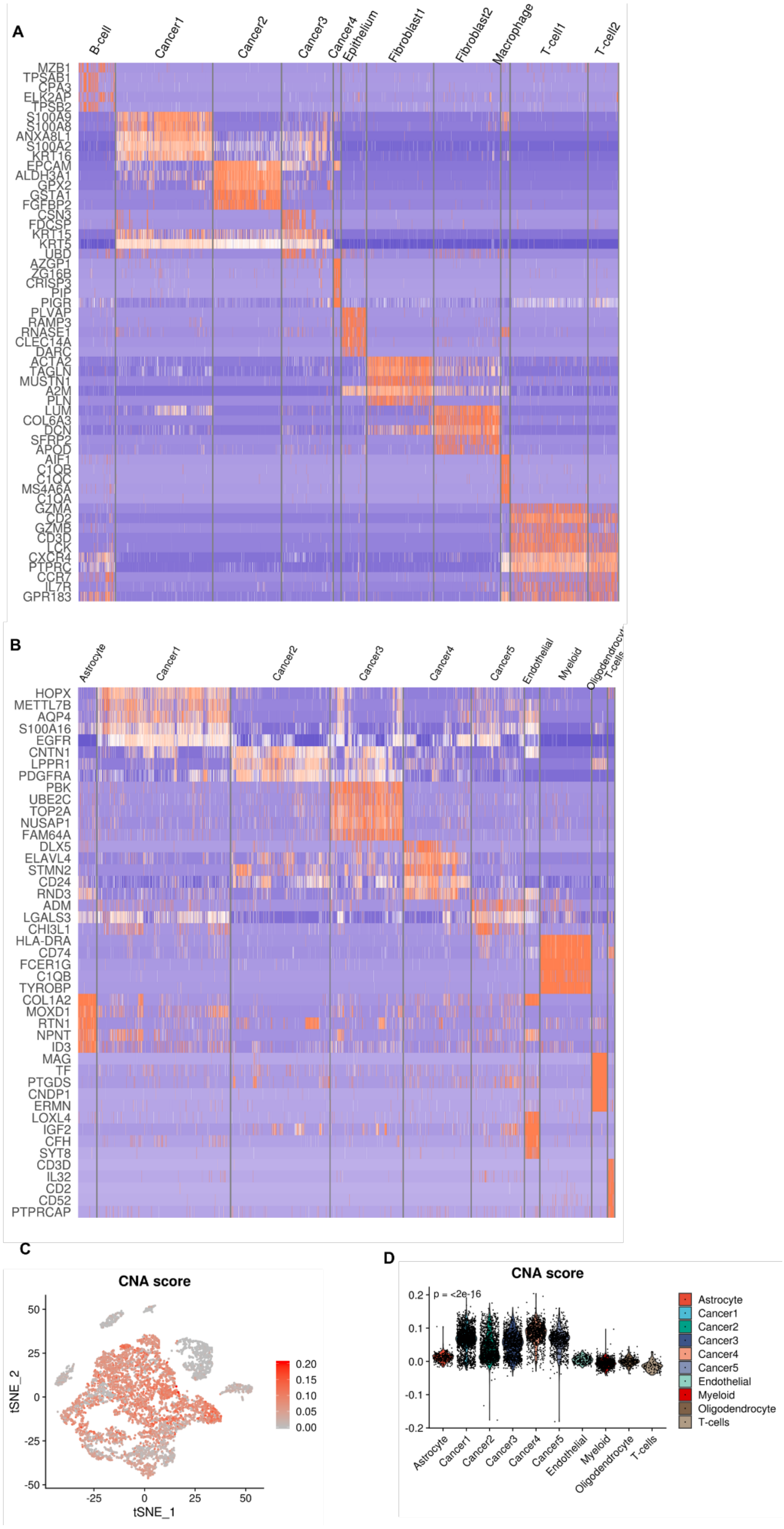

A
B-cell
Cancer1
Cancer2
Cancer3
Cancer4
Epithelium
Fibroblast1
Fibroblast2
Macrophage
T-cell1
T-cell2
MZB1
TPSAB1
CPA3
ELK2AP
TPSB2
S100A9
S100A8
ANXA8L1
S100A2
KRT16
EPCAM
ALDH3A1
GPX2
GSTA1
FGFBP2
CSN3
FDCSP
KRT15
KRT5
UBD
AZGP1
ZG16B
CRISP3
PIP
PIGR
PLVAP
RAMP3
RNASE1
CLEC14A
DARC
ACTA2
TAGLN
MUSTN1
A2M
PLN
LUM
COL6A3
DCN
SFRP2
APOD
AIF1
C1QB
C1QC
MS4A6A
C1QA
GZMA
CD2
GZMB
CD3D
LCK
CXCR4
PTPRC
CCR7
IL7R
GPR183
B
Astrocyte
Cancer1
Cancer2
Cancer3
Cancer4
Cancer5
Endothelial
Myeloid
Oligodendrocyte
T-cells
HOPX
METTL7B
AQP4
S100A16
EGFR
CNTN1
LPPR1
PDGFRA
PBK
UBE2C
TOP2A
NUSAP1
FAM64A
DLX5
ELAVL4
STMN2
CD24
RND3
ADM
LGALS3
CHI3L1
HLA-DRA
CD74
FCER1G
C1QB
TYROBP
COL1A2
MOXD1
RTN1
NPNT
ID3
MAG
TF
PTGDS
CNDP1
ERMN
LOXL4
IGF2
CFH
SYT8
CD3D
IL32
CD2
CD52
PTPRCAP
C
CNA score
50
25
0
-25
-50
tSNE_2
-25
0
25
50
tSNE_1
0.20
0.15
0.10
0.05
0.00
D
CNA score
p = <2e-16
0.2
0.1
0.0
-0.1
-0.2
Astrocyte
Cancer1
Cancer2
Cancer3
Cancer4
Cancer5
Endothelial
Myeloid
Oligodendrocyte
T-cells

Supplementary Figure 3. Clustering analysis of single-cell RNA sequencing data of head and neck squamous cell carcinoma and glioblastoma multiforme. (A) Heatmap showing the top five genes for each cluster by single-cell RNA sequencing analysis of head and neck squamous cell carcinoma. (B) Heatmap showing the top five genes for each cluster by single-cell RNA sequencing analysis of glioblastoma multiforme. (C) t-SNE plot showing CNA score across cancer and immune cell clusters of glioblastoma multiforme. (D) Violin plot showing CNA score of cancer and immune cell clusters. (CNA: chromosome copy number alteration)

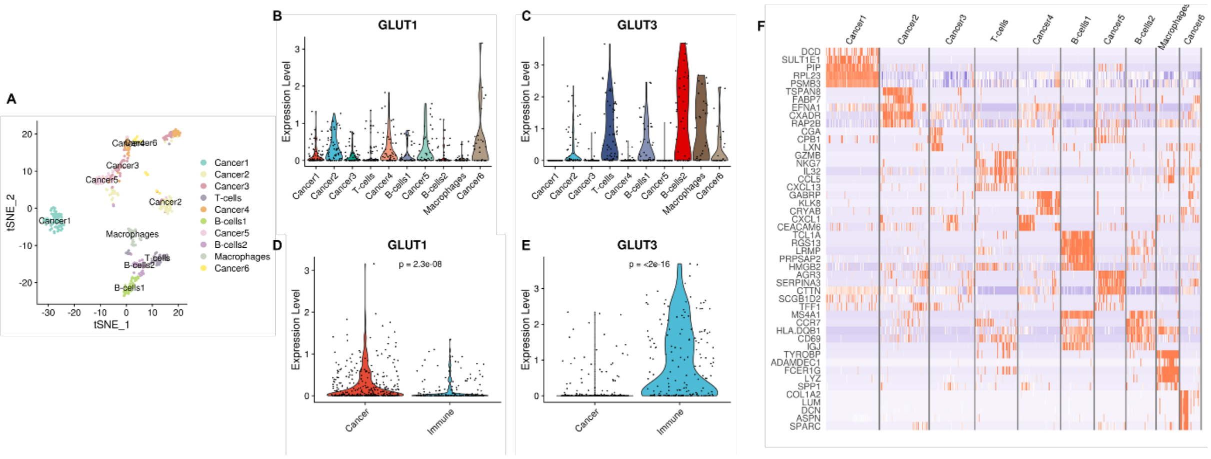

Supplementary Figure 4. Distribution of GLUT1 and GLUT3 within the tumor microenvironment of breast cancer. (A) Two-dimensional visualization of single cell transcriptome data of breast cancer by t-SNE analysis. (B, C) Violin plots showing the expression distribution of GLUT1 (*left*) and GLUT3 (*right*) across cancer and stromal cell clusters of breast cancer. (D, E) Violin plots showing GLUT1 and GLUT3 expression in cancer and immune cell clusters. (F) Heatmap showing the top five genes for each cluster of breast cancer

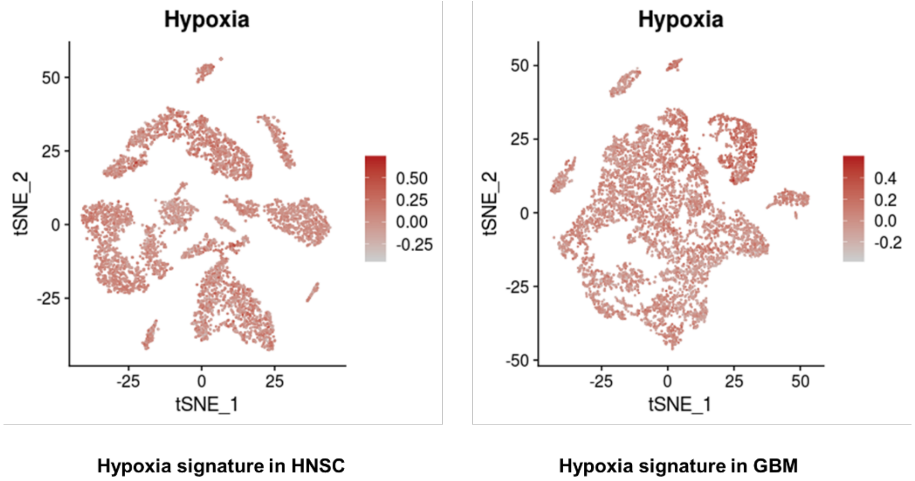

Supplementary Figure 5. Hypoxia signature in tumor microenvironment. The hypoxia score was estimated by scRNA-seq data of HNSC and GBM. While GLUT1 and GLUT3 were differently expressed in cancer and immune cell subtypes, the hypoxia score was not specifically upregulated in a certain cell type.

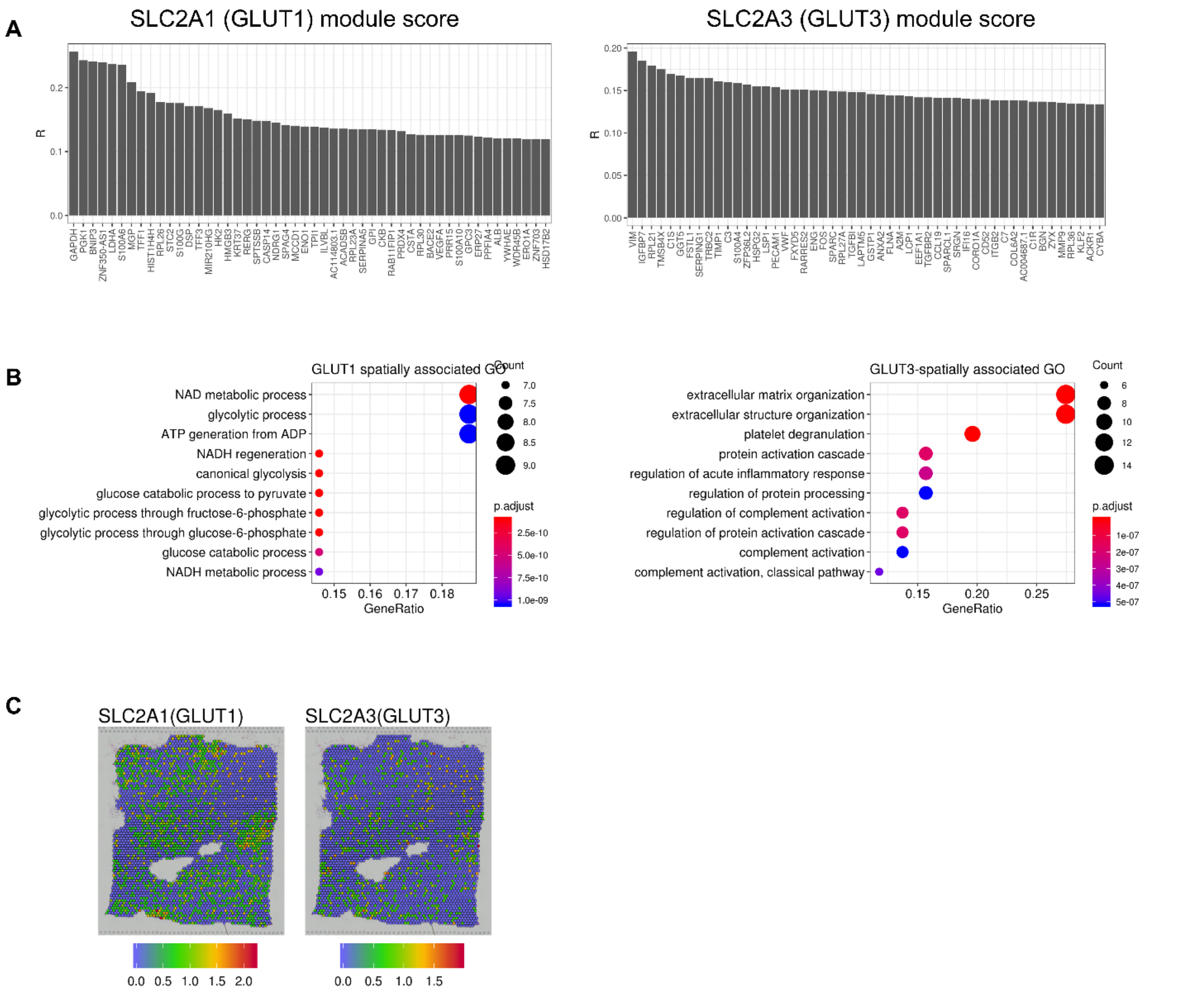

Supplementary Figure 6. Modules spatially associated with GLUT1 and GLUT3. (A) GLUT1 and GLUT3 module scores were evaluated using positively correlated genes with SLC2A1 (GLUT1. *left*) and SLC2A3 (GLUT3, *right*). Top-50 genes that were positively correlated with GLUT1 and GLUT3 were plotted, respectively. (B) The gene ontology of correlated genes with GLUT1 and GLUT3 expression was represented. (C) The spatial distribution of GLUT1 and GLUT3 expression overlaid with breast cancer tissue was depicted.

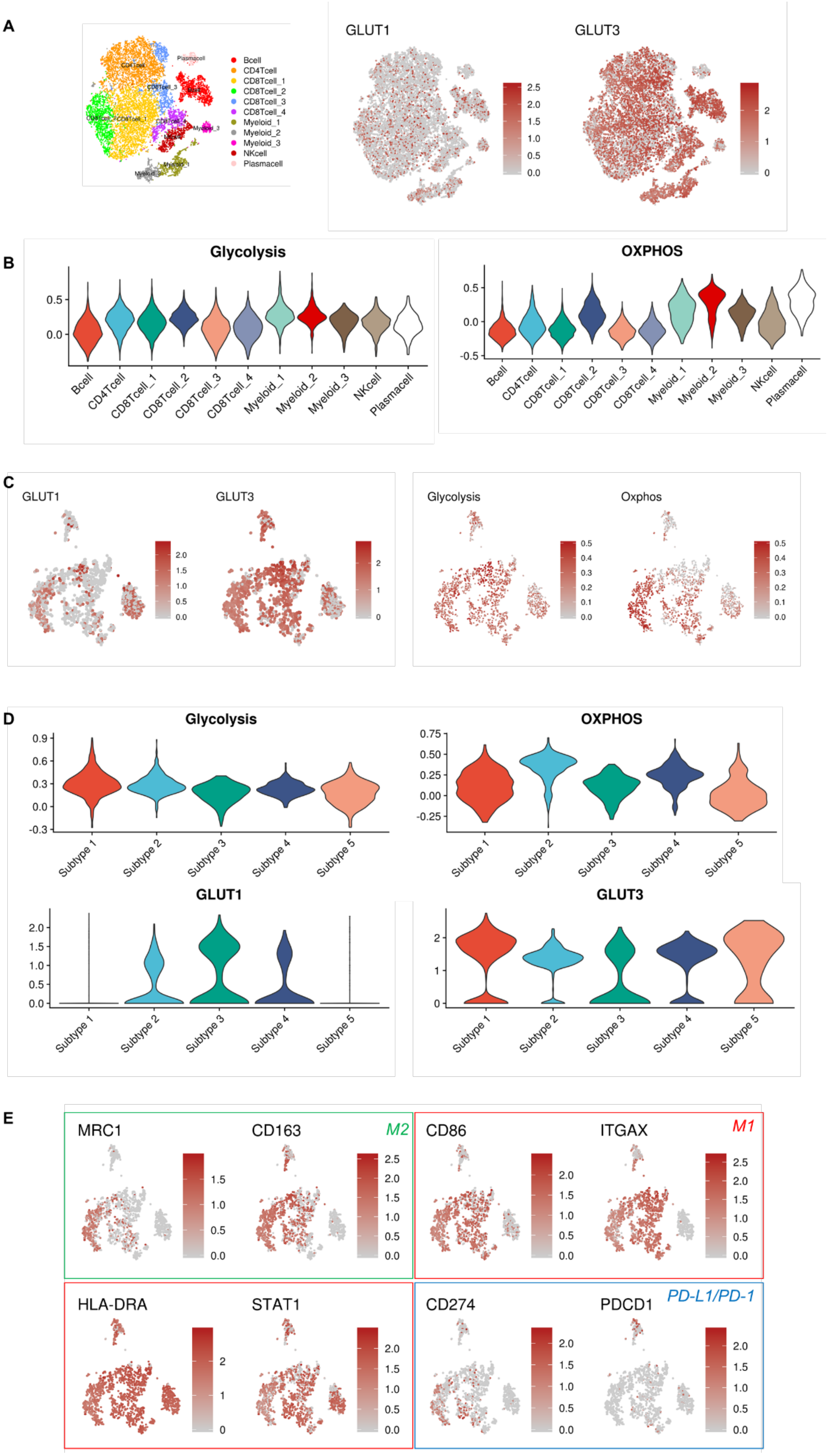
A
Bcell
CD4Tcell
CD8Tcell_1
CD8Tcell_2
CD8Tcell_3
CD8Tcell_4
Myeloid_1
Myeloid_2
Myeloid_3
NKcell
Plasmacell
GLUT1
GLUT3
B
Glycolysis
OXPHOS
C
GLUT1
GLUT3
Glycolysis
Oxphos
D
Glycolysis
OXPHOS
GLUT1
GLUT3
Subtype 1
Subtype 2
Subtype 3
Subtype 4
Subtype 5
E
MRC1
CD163
M2
CD86
ITGAX
M1
HLA-DRA
STAT1
CD274
PDCD1
PD-L1/PD-1

Supplementary Figure 7. Distribution of glucose transporter and metabolic activity of immune cells of melanoma TME. (A) t-SNE plot showing immune cell clusters of melanoma patients and the different expression pattern of GLUT1 and GLUT3 across these clusters. (B) Violin plot showing glycolysis and OXPHOS activity scores across 11 immune cell clusters. (C) t-SNE plots indicating the different patterns of GLUT1 and GLUT3, and the glycolytic and OXPHOS activity scores across myeloid subtype clusters of melanoma patients. (D) Violin plots showing GLUT1 and GLUT3 expressions and the glycolytic and OXPHOS activity scores across five myeloid subtypes of human melanoma samples. (E) t-SNE plots showing expression patterns of typical markers of M1 (CD86, ITGAX, HLA-DRA, STAT1) and M2 (MRC1, CD163) macrophage, PD-L1, and PD-1 across myeloid subtypes of human melanoma samples.

1.2 Supplementary Tables

Supplementary Table 1. Summary of Deposited Data

Deposited Data	SOURCE	IDENTIFIER
RNA-seq of human head and neck carcinoma samples	(Puram et al., 2017)	GSE103322
RNA-seq of human glioblastoma multiforme	(Neftel et al., 2019)	GSE131928
RNA-seq of human breast cancer	(Chung et al., 2017)	GSE75688
RNA-seq of human melanoma samples	(Sade-Feldman et al., 2018)	GSE120575
RNA sequencing files from all cancers from TCGA projects	The Cancer Genome Atlas	portal.gdc.cancer.gov
Spatial transcriptome data of breast cancer samples	https://www.10xgenomics.com/resources/datasets/	https://www.10xgenomics.com/resources/datasets/
